# 92. System-wide Implementation of a New Urine Culture to Optimize Diagnostic Stewardship

**DOI:** 10.1093/ofid/ofad500.008

**Published:** 2023-11-27

**Authors:** Lisa Parlich, Raymond Gunn, Asra Salim, Bill Eberlein, Christina Silkaitis

**Affiliations:** Northwestern Medicine, Woodstock, IL; Northwestern Medicine, Woodstock, IL; Northwestern Memorial Healthcare, Chicago, Illinois; Northwestern Medicine, Woodstock, IL; Northwestern Medicine, Woodstock, IL

## Abstract

**Background:**

Our healthcare system consists of 8 acute care facilities, each of which utilizes a Urinalysis (UA) with Reflex to Culture laboratory order. With this order, a urine culture is performed automatically if the UA flags as abnormal, thus resulting in overutilization of urine cultures and subsequent identification of urinary tract infections (UTI), including hospital-associated catheter UTI (CAUTI).

**Methods:**

The two largest facilities developed and implemented a new urine order to ensure appropriate utilization of urine cultures on adult critical care (CC) patients: the Urinalysis with Hold for Culture (UA/Hold) order. With this order, a culture would not be completed until a clinician reviewed the UA results, evaluated the need for a culture and then placed an add-on urine culture within 48 hours of the original UA specimen. If the UA was not abnormal, an override reason was chosen for the order, i.e. new urgency, frequency, dysuria, fever, altered mental status, hematuria. Logic existed within this order to not allow a urine culture unless a UA had been performed within the last 48 hours or if a patient was neutropenic, pregnant or undergoing a urological procedure.

**Results:**

From February 2021 to February 2022, the two facilities who implemented the order avoided a total of 485 cultures (Table 1). These two sites also experienced a decrease in CAUTIs compared to the year prior to implementation. The UA/Hold order resulted in a meaningful reduction in CAUTI rates since urine culture orders were only placed on patients that clinically needed them.

Due to the success of the new order, it was expanded to five additional facilities and their CC patient populations in December 2022. Data collected January-March 2023, demonstrates the effectiveness of the implementation, as zero CAUTIs have been reported from CC patients at any of the five hospitals. In the 12 months prior to implementation, 21 CAUTIs had been reported. Overall, 27 cultures were avoided (Table 2).

**Figure:**
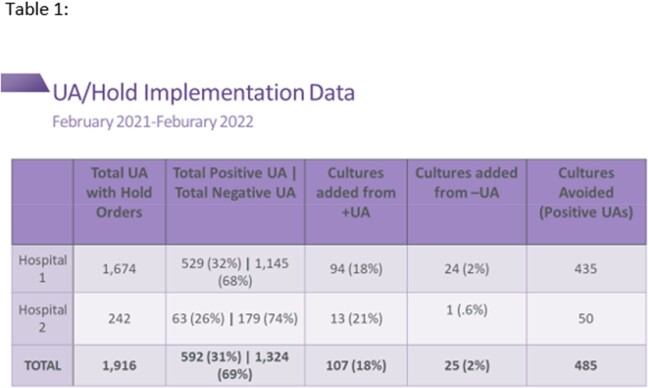

**Figure:**
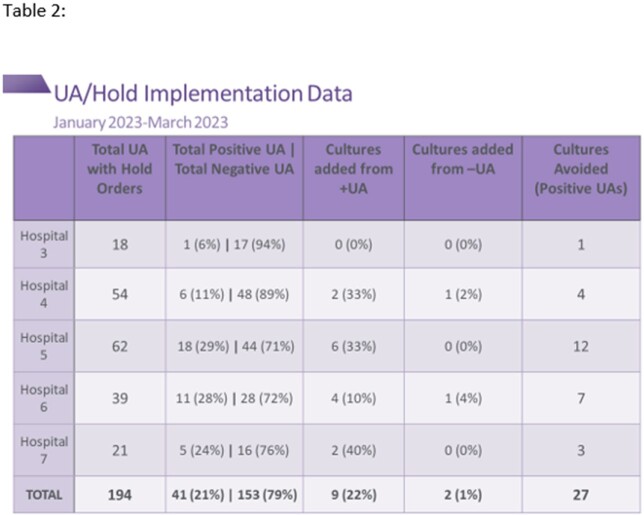

**Conclusion:**

With exceptional results from the first three months since implementation, the months to come show promise for significant culture and CAUTI reduction across the system and provides opportunity to expand the order to non-critical care units as well.

**Disclosures:**

**All Authors**: No reported disclosures

